# Activating transcription factor 4-dependent hsa-miR-663a transcription mediates mTORC1/p70S6K1 signaling underleucine deprivation

**DOI:** 10.3389/fnut.2022.965771

**Published:** 2022-08-05

**Authors:** Junki Yamamura, Sihui Ma, Huijuan Jia, Hisanori Kato

**Affiliations:** Health Nutrition, Graduate School of Agricultural and Life Sciences, The University of Tokyo, Tokyo, Japan

**Keywords:** ATF4, hsa-miR-663a, leucine deprivation, microRNAs, mTORC1

## Abstract

The mechanistic target of rapamycin complex 1 (mTORC1) is involved in nutrient-induced signaling and is a master regulator of cell growth and metabolism. Amino acid-deficient conditions affect mTORC1 activity; however, its upstream regulators warrant further investigation. MicroRNAs are key regulators of nutrient-related responses; therefore, the present study aimed to assess the leucine starvation-induced microRNA profile and its impact on mTORC1 activity. Transcriptome analysis of human hepatocellular carcinoma cells (HepG2) under leucine deprivation revealed that hsa-miR-663a and hsa-miR-1469 were altered in a transcription factor 4-dependent manner. Overexpression of these microRNAs induced phosphorylation of the ribosomal protein S6 kinase beta-1, a mTORC1 downstream target. Furthermore, hsa-miR-663a downregulated proline-rich Akt1 substrate of 40 kDa (PRAS40), one of the mTORC1 components. In summary, this study provides new insights into the regulatory role of microRNAs in amino acid metabolism and demonstrates alterations in microRNA profile under leucine deprivation in human hepatocytes.

## Introduction

Malnutrition can lead to chronic health conditions and the development of various pathologies, such as obesity, diabetes, and cardiac disorders (1). Adequate dietary intake of essential amino acids (EAA) is vital for health, while insufficient EAA intake is associated with disease progression. Recent development has linked inadequate EAA intake with abnormal regulatory patterns of the microRNAs (miRNAs) secretion network (2, 3). Among the nine proposed EAAs, leucine has been extensively reported for its crucial role in cellular growth and cell cycle regulation (4, 5). Depletion of leucine is also associated with caspase-dependent apoptosis, amino acid (AA) metabolism, and other vital cellular events (6).

Amino acid levels are finely controlled in cells by balancing their synthesis, uptake, and utilization (7). Eukaryotes have developed two opposite regulatory mechanisms for AA sensing: the mechanistic mammalian target of rapamycin complex 1 (mTORC1) signaling pathway, which is regulated during AA abundance, and the general control non-derepressible 2 (GCN2) signaling pathway, which is activated under AA deficiency (8). The mTORC1 signaling pathway is regulated by various stimulators. When circulating AA levels are adequate, mTORC1 is activated and promotes the synthesis of several downstream proteins by phosphorylating eukaryotic translation initiation factor 4E (eIF4E)-binding proteins, S6 kinases, and the eukaryotic elongation factor 2 kinase to favor protein synthesis and sustain cell function. In contrast, when circulating AAs are inadequate, the GCN2 signaling pathway is activated owing to the accumulation of uncharged transfer RNAs. This, in turn, promotes the phosphorylation of the eukaryotic initiation factor 2 alpha (eIF2α), with consequent global repression of translation. Nevertheless, it induces the production of specific mRNAs, such as the activating transcription factor 4 (ATF4), the master regulator of several AA synthases and transporters (9). The above regulatory systems function through AA-sparing effects, thus ensuring a constant supply of AA and maintaining metabolic homeostasis (10).

MiRNAs, a class of single-stranded non-coding RNAs comprising 22–26 nucleotides, target the 3′-untranslated region (3′-UTR) of mRNAs to post-transcriptionally induce their inhibition and degradation (11). The biosynthesis of miRNAs occurs in the nucleus by RNA polymerase II in the same manner as that of the protein-coding mRNAs. There are mainly two reported pathways of miRNA biosynthesis: the canonical pathway and the mirtron pathway (12). One-third of the human genome is estimated to be regulated by miRNAs, and their dynamic expression behaviors in different organs are associated with various pathologies of diseases, such as cancer, diabetes, and sarcopenia (13–15). Several lines of evidence suggest that miRNAs may also regulate pathways involved in AA metabolism (16). For instance, miR-29b targets the branched-chain α-ketoacid dehydrogenase complex in mammalian cells, consequently regulating the branched-chain AA metabolism (17). However, how miRNAs are involved in the biological response to AA starvation *via* the classic mTORC1/GCN2 pathway remains to be elucidated. Therefore, the present study aimed to investigate the miRNA profile under leucine deprivation and its relationship with the mTORC1/GCN2 signaling pathway using *in vitro* models.

## Materials and methods

### Cell culture and treatments

Human hepatocellular carcinoma cells (HepG2) were obtained from the American Type Culture Collection (Rockville, MD, United States) and cultured in Dulbecco’s modified Eagle’s medium (DMEM; Sigma-Aldrich, St. Louis, MO, United States) containing 10% (v/v) fetal bovine serum (Sigma-Aldrich) and 1% (v/v) penicillin-streptomycin (Sigma-Aldrich) at 37°C and 5% (v/v) CO_2_ atmosphere. For leucine starvation, the cells were grown in minimum essential medium (Sigma-Aldrich) lacking leucine but supplemented with 10% (v/v) fetal bovine serum. Before starvation, the cells were rinsed twice with phosphate-buffered saline (Gibco, Waltham, MA, United States). All experiments were repeated at least three times.

### Plasmid construction

Template cDNA for *ATF4* subcloning was prepared from total RNA isolated from HepG2 cells using the PrimeScript RT Master Mix (Takara Bio, Kusatsu, Japan) following the manufacturer’s instructions. The open reading frame of *ATF4* (1,088 bp) was obtained by polymerase chain reaction (PCR) using primers containing *Bam*HI or *Eco*RI restriction sites at their 5′-end. The sequences of the forward and reverse primers were 5′–ACAGGATCCATTCCAGCAAAGCA–3′ and 5′–CTAGAATTCTCTATGTACAAGCACA–3′, respectively. Amplified fragments were then cloned into the *Bam*HI and *Eco*RI restriction sites of pcDNA3.1 (+) Mammalian Expression Vector (Thermo Fisher Scientific, Waltham, MA, United States).

### Activating transcription factor 4 overexpression and knockdown

HepG2 cells were seeded on 6-well plates at 70–80% confluent density and cultured for 24 h in antibiotic-free DMEM. For overexpression experiments, *ATF4*-overexpressing plasmid and pcDNA3.1 empty vector were transfected into HepG2 cells using Invitrogen Lipofectamine 2000 (Thermo Fisher Scientific) following the manufacturer’s instructions. After 6 h, the culture medium was replaced with a complete medium and incubated for 24 h. For knockdown experiments, small interference RNAs (siRNAs) targeting *ATF4* (cat# SI03019) were purchased from Qiagen (Hilden, Germany). Following the manufacturer’s instructions, the siRNA was transfected into HepG2 cells using Invitrogen Lipofectamine RNAi max transfection reagent (Thermo Fisher Scientific). After 6 h, the culture medium was substituted with minimum essential medium containing or lacking leucine and cultured for 12 h.

### MicroRNA overexpression

HepG2 cells were plated on 6-well plates at 70–80% density and cultured for 24 h in antibiotic-free DMEM. miRNA mimics (cat# MIM0663, MIM0829, and MIM9001) were purchased from Active Motif (Carlsbad, CA, United States) and transfected into HepG2 cells using Invitrogen Lipofectamine RNAi max transfection reagent (Thermo Fisher Scientific) following the manufacturer’s instructions. After 6 h, the culture medium was replaced with DMEM for 48 h.

### Total RNA extraction, microRNA microarray analysis, and polymerase chain reaction analysis

Total RNA was extracted from HepG2 cells using TRIzol reagent (Thermo Fisher Scientific). For global miRNA expression profiling, total RNA (1,000 ng) was labeled using the FlashtagTM RNA Labeling Kit (Genisphere, Hatfield, PA, United States) and hybridized with the miRNA 3.0 array (Affymetrix, Santa Clara, CA, United States) following the manufacturer’s instructions. After washing and staining, array scanning was performed using GeneChip Hybridization and Affymetrix Gene Array Scanner 3000 (Affymetrix), with a threshold set as log ratio > 0.6 compared with control. For *ATF4* expression analysis, real-time PCR primers were designed using PRIMER3^1^ (18). The relative expression of *ATF4* was determined relative to that of *GAPDH* and is shown as fold-change compared with the Mock group. To analyze hsa-miR-663a and hsa-miR-1469, primers were purchased from QIAGEN (Hs_miR-663_3 miScript Primer Assay, MS00037247; Hs_miR-1469_2 miScript Primer Assay, MS00037800; and Hs_RNU6-2_11 miScript Primer Assay, MS00033740). miRNA expression was normalized against *U6* expression.

### Immunoblot analysis

ATF4 (#11815), PRAS40 (#2691), Ras (#3339), phospho-p70 S6 kinase (#9205), p70 S6 kinase (#2708), and β-actin (#3700) antibodies were purchased from Cell Signaling Technology (Danvers, MA, United States). GAPDH (sc-20357) antibody was obtained from Santa Cruz Biotechnology (Dallas, TX, United States). Normal mouse IgG (sc-2027) antibodies were purchased from GE Healthcare (Chicago, IL, United States). Total proteins were extracted from HepG2 cells using cold radioimmunoprecipitation assay (RIPA) lysis buffer with a proteinase inhibitor and separated by centrifugation at 12000 × *g* for 30 min at 4°C. Protein concentration was measured using Bio-Rad Protein Assay (Bio-Rad, Hercules, CA, United States). Equivalent amounts of total proteins were separated using sodium dodecyl-sulfate-polyacrylamide gel electrophoresis and transferred electrophoretically onto Hybond P polyvinylidene difluoride (PVDF, GE Healthcare) membranes, which were incubated for 1 h at room temperature with PVDF Blocking Reagent for Can Get Signal (TOYOBO, Osaka, Japan), followed by washing with wash buffer (1 × Tris-buffered saline with 0.01% Tween-20). The membranes were then incubated overnight at 4°C with a 1:1000 dilution of primary antibodies followed by incubation with a 1:5000 dilution of secondary antibodies for 1 h. The membranes were washed with wash buffer after each step. Finally, the membranes were incubated with the ECL Western Blotting Detection System (GE Healthcare). Protein levels were detected by chemiluminescence using Ez-Capture MG (ATTO, Tokyo, Japan).

### MicroRNA target prediction

Target gene candidates of hsa-miR-663a and hsa-miR-1469 were extracted using the Cumulative weighted context++score in the TargetScan Human 7.0 database^2^ (19). Among 593 genes that showed decreased expression (log ratio < 0.1 vs. control) under leucine deprivation, the top five genes showing high scores were extracted.

### Statistical analysis

The results are presented as mean ± standard error (SE). Data were analyzed using one-way and two-way analysis of variance in Microsoft Excel (Microsoft Corporation, Redmond, WA, United States). Significant differences between groups were evaluated using Dunnett’s test except for the validation of the relationship between ATF4 and hsa-miR-663a and hsa-miR-1469. In these analyses, Student’s *t*-test was employed to determine the differences between Control and Leu (-) in ATF4(+) or ATF4(-) conditions. The significance level was set at *p* < 0.05.

## Results

### Leucine deprivation or activating transcription factor 4 overexpression alters the microRNA profile

**FIGURE 1:**
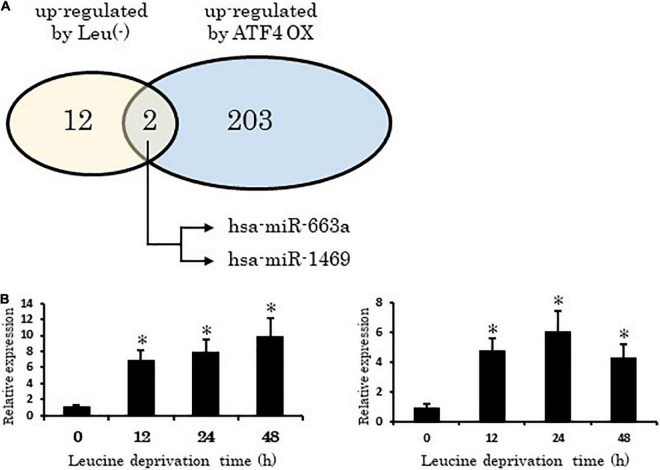
Activating transcription factor 4 (ATF4) directly regulates microRNAs under leucine deprivation. **(A)** Two miRNAs, hsa-miR-663a and hsa-miR-1469, show increased expression in ATF4 overexpression and leucine deprivation experiments. **(B)** Time-course expression of hsa-miR-663a (left) and hsa-miR-1469 (right). Leu (-), leucine deprivation; ATF4 OX, activating transcription factor 4 overexpression. Data are shown as means ± standard error (SE) of three independent experiments. *p < 0.05 vs. Control.

A total of 21,118 human miRNAs were assessed using the Affymetrix GeneChip. Among the altered miRNAs, 12 were found to be upregulated by leucine deprivation. Our analysis also indicated that 203 miRNAs were upregulated (log ratio > 0.6 compared with control, data not shown) under *ATF4* overexpression (Figure 1A and Supplementary Figure 1). Among the upregulated miRNAs identified, hsa-miR-663a and hsa-miR-1469 consistently showed increased expression in leucine deprivation and *ATF4* overexpression conditions. Furthermore, in a time-course analysis, the expression of both miRNAs significantly increased 12 h after leucine deprivation (Figure 1B).

### Activating transcription factor 4 directly regulates hsa-miR-663a and hsa-miR-1469

**FIGURE 2:**
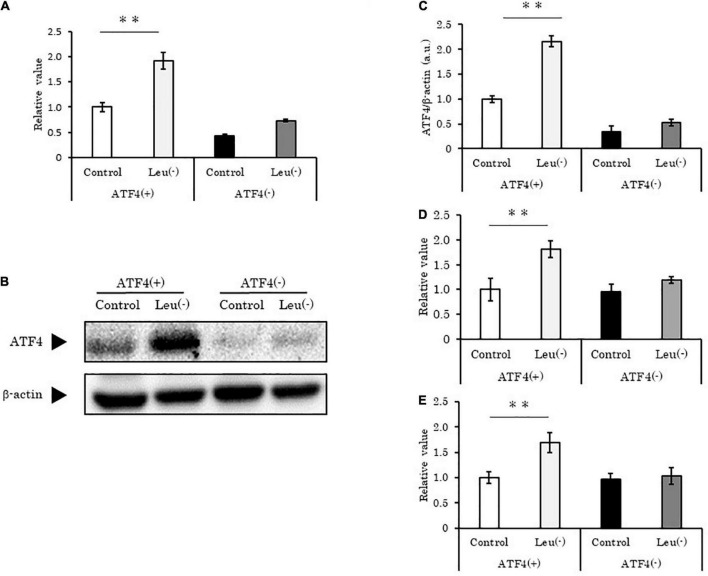
Activating transcription factor 4 knockdown suppresses hsa-miR-663a and hsa-miR-1469 induction under leucine deprivation in HepG2 cells. **(A)** HepG2 cells transfected with *ATF4*-specific siRNA for 12 h, followed by 12 h of leucine deprivation, and then analyzed using PCR for *ATF4* mRNA level. **(B)** HepG2 cells were treated as in panel **(A)** and analyzed using western blotting for ATF4 and β-actin protein levels. **(C)** Quantification of ATF4/β-actin as described in panel **(B)**. **(D,E)** HepG2 cells were treated as in panel **(A)** and analyzed using PCR for **(D)** hsa-miR-663a and **(E)** hsa-miR-1469 levels. Data are mean ± SE of three independent experiments. *p < 0.05 and **p < 0.01, vs. Control. Leu (-), leucine deprivation; ATF4, activating transcription factor 4; ATF4(+), HepG2 cell with normal expression of ATF4; ATF4(-), HepG2 cell with *ATF4* expression knocked down.

To validate the relationship between ATF4 and the two upregulated miRNAs, we knocked down *ATF4* in HepG2 cells using targeted siRNAs. Upon introduction of *ATF4*-specific siRNA, ATF4 mRNA and protein levels were significantly reduced (*p* < 0.01 vs. control) to amounts lower than those observed in leucine deprivation condition (Figures 2A–C). Concomitantly, hsa-miR-663a and hsa-miR-1469 showed the same behavior as *ATF4* (Figures 2D,E). ATF4 is reported to act as a transcription factor and is considered to bind to specific sites in the DNA. Identical or similar specific sites, AARE and CRE (TTACATCAT and TGACGTCA, respectively; data not shown), were found in the upstream region of the host genes of these miRNAs. Taken together, we concluded that ATF4 directly regulates these miRNAs under leucine deprivation.

### Discovery of candidate targets of hsa-miR-663a and hsa-miR-1469

To identify the specific target genes of the two identified miRNAs, we conducted an integrated analysis using the TargetScanHuman 7.0 database based on the transcriptomic data obtained from HepG2 cells after 48 h of leucine deprivation. Among 593 genes that were regulated under leucine deprivation, 97 and 62 genes contained binding sites in their 3′-UTR for hsa-miR-663a and hsa-miR-1469, respectively. The analysis revealed that *AKT1S1* encoding proline-rich Akt1 substrate of 40 kDa (PRAS40) showed a high score among all genes for both miRNAs, while the score was the highest for hsa-miR-663a (Supplementary Table 1). Therefore, our subsequent experiments focused on the interaction between hsa-miR-663a/hsa-miR-1469 and PRAS40.

### PRAS40 is a direct target of hsa-miR-663a

**FIGURE 3:**
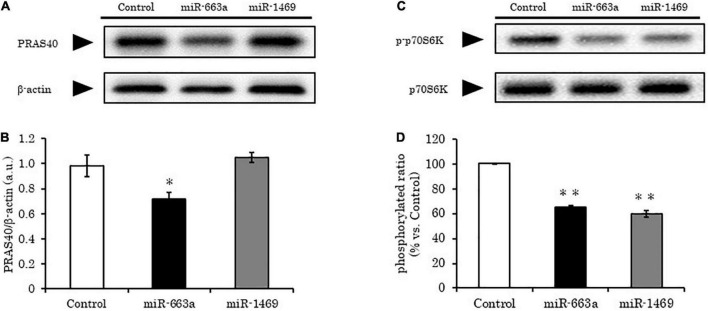
hsa-miR-663a targeting PRAS40 and hsa-miR-1469 decrease phosphorylated p70S6K levels in HepG2 cells. HepG2 cells were transfected with 80 nM hsa-miR-663a and hsa-miR-1469 mimics for 48 h and analyzed using western blotting. **(A)** Representative western blot bands for PRAS40. **(B)** Quantification of panel **(A)**. **(C)** Representative western blot bands for p70S6K and phosphorylated p70S6k. **(D)** Quantification of panel **(C)**. Data are mean ± SE. *p < 0.05 and **p < 0.01, vs. Control.

PRAS40 acts at the intersection of Akt- and mTOR-mediated signaling pathways. Two binding sites were predicted for hsa-miR-663a on PRAS40 3′-UTR (Supplementary Table 2). To confirm whether hsa-miR-663a truly targeted PRAS40, we evaluated PRAS40 levels in HepG2 cells overexpressing hsa-miR-663a. Compared to the control, PRAS40 expression significantly decreased (20–30%) after 48 h of hsa-miR-663a overexpression (*p* < 0.01), suggesting that hsa-miR-663a directly targets PRAS40. In contrast, PRAS40 levels remained similar to that in control despite overexpression of hsa-miR-1469, suggesting that PRAS40 may not be a direct target of hsa-miR-1469 (Figures 3A,B).

### hsa-miR-663a decreases mTORC1 activity by negatively regulating PRAS40

As PRAS40 inhibits mTORC1 activity, we expected that hsa-miR-663a overexpression would increase mTORC1 activity. However, the levels of phosphorylated p70S6K (a marker of mTORC1 activity) were significantly decreased (35%) in HepG2 cells overexpressing hsa-miR-663a compared to that in control (*p* < 0.01) (Figures 3C,D). These results were consistent with another study that reported decreased mTORC1 activity upon PRAS40 knockdown (20). Based on these findings, we concluded that hsa-miR-663a controls mTORC1 activity by regulating PRAS40 levels.

## Discussion

Amino acids are not only essential for nutrition, but can also act as signaling molecules. AA levels within organs can be sensed and accurately transduced to downstream signals vital for cells and organisms. As mentioned in the Introduction, the AA sensing machinery is mediated by: (1) the mTORC1 pathway, which regulates cell growth upon AA abundancy, and (2) the GCN pathway, which contributes to AA conservation and promotes AA recycling. Under AA insufficiency, the activation of the mTORC1-dependent pathway rapidly inhibits the phosphorylation of p70S6K and eIF4E, thereby inactivating mRNA translation for AA conservation (21). Reciprocally, specific transcription factors, such as ATF4, are concurrently activated, initiating the expression of specific genes (e.g., asparagine synthetase) to adapt to the corresponding situation (22). This pathway named “general amino acid control” is crucial for cell survival under AA deprivation.

MicroRNAs have emerged as key regulators of cellular homeostasis over the past decade, and their aberrant expression induced by environmental stimulus has been shown to be associated with many metabolic phenomena. Dietary interventions can modify the miRNA profile, which in turn will regulate target mRNAs at the transcriptional/translational levels. Overall, these regulatory mechanisms ensure cellular homeostasis. Previous studies have reported that miRNA profiles were altered under leucine deprivation in murine models, and several miRNAs have been reported to be regulated (23–25). For instance, inhibition of miR-215-5p reversed the suppressive effects of lipogenesis genes induced by leucine deficiency in murine hepatocytes (23). miR-20a and miR-106b negatively regulate autophagy-relating gene expression in murine myoblasts (24). In the present study, we identified two miRNAs (hsa-miR-663a and hsa-miR-1469) that were upregulated by both ATF4 signals and leucine deprivation.

hsa-miR-663a was previously implicated in immune response regulation and has shown to be associated with several disorders and diseases, including systematic lupus erythematosus and ovarian cancer (25). hsa-miR-1469 was reported to be p53-responsive; thus, its downregulation may promote cancer cell migration and invasion (26). In particular, among its related pathways, the nuclear receptor transcription pathway and the regulation of lipid metabolism by peroxisome proliferator-activated receptor alpha are often described (26). Nevertheless, the detailed function of these two miRNAs remains unclear, and our report is the first to suggest an interaction between them and the mTORC1/GCN pathways.

Under nutrient starvation, cells generally decrease their proliferation to adapt and survive. In agreement with this behavior, our study demonstrated that leucine-deprivation induced miRNA profile alterations, which consequently downregulated the phosphorylation of p70S6K, a critical marker of cellular growth regulation. Furthermore, the findings also revealed that hsa-miR-663a regulated mTORC1 activity by targeting PRAS40, a component of the mTORC1 complex (27). PRAS40 dissociates from mTORC1 through phosphorylation by Akt in the presence of insulin, thereby inducing mTORC1 to change into its active form; thus, PRAS40 is considered an inhibitor of mTORC1 (28). However, Hong-Brown et al. (20) reported that PRAS40-knockdown reduces mTORC1 activity and prevents phosphorylation of its downstream target p70S6K in mouse myotubes. Considering the findings of the present and previous studies, we inferred that the inhibition of p70S6K activity observed in hsa-miR-663a-overexpressing HepG2 cells in this study was derived from the downregulation of PRAS40.

Previous studies have also shown that p70S6K induces insulin resistance *via* phosphorylation of serine residues (Ser-302/307, Ser-307/312, Ser-632/636, and Ser-1097/1101) of the insulin receptor subtract 1 and subsequent attenuation of the PI3K/AKT pathway (29). For instance, Xiao et al. (4) reported that leucine deprivation enhanced insulin sensitivity in the liver and hepatocytes of mice through the downregulation of p70S6K following reduced GCN2 phosphorylation. In the present study, we showed that hsa-miR-663a, which is induced by the GCN2/ATF4 pathway under leucine deprivation, decreased p70S6K phosphorylation. Hence, our results are consistent with previous reports and suggest hsa-miR-663a as a novel effector between GCN2 and mTORC1 pathways.

Our study has some limitations. We could not identify the target gene of hsa-miR-1469 despite its ability to inhibit p70S6K. Although PRAS40 contained one target site of this miRNA, PRAS40 levels were not affected by hsa-miR-1469 overexpression. Therefore, further omics-based study is encouraged to identify the target gene of hsa-miR-1469. Furthermore, our analysis identified some additional miRNAs (hsa-miR-149-3p, hsa-miR-762, and hsa-miR-3169) that were also upregulated in leucine-deprived HepG2 cells (data not shown), which could also target PRAS40. Thus, other than hsa-miR-663a, PRAS40 expression may be regulated by multiple miRNAs at the same time.

## Conclusion

**FIGURE 4:**
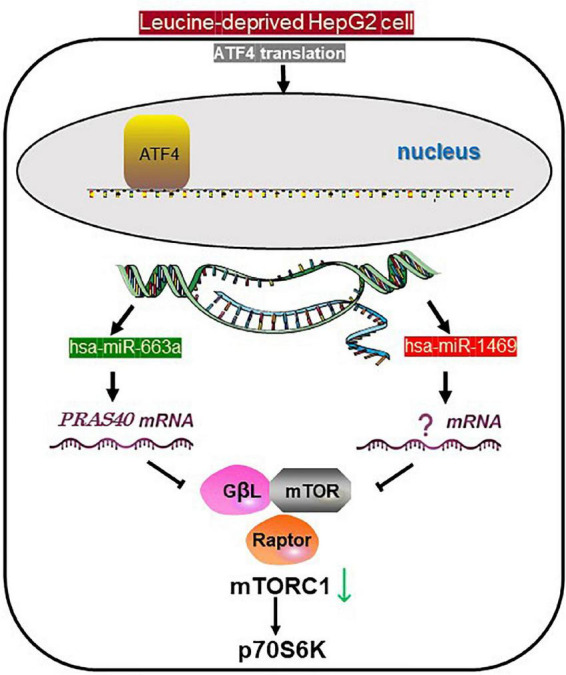
Proposed schematic for the role of the miRNAs under leucine starvation. In leucine-deprived HepG2 cells, activated transcription of hsa-miR-663a and hsa-miR-1469 is induced by ATF4, specifically translated *via* GCN2/eIF2a. Both miRNAs induce the downregulation of phosphorylated p70S6K. However, only hsa-miR-663a targets PRAS40, one component of mTORC1, and inhibits mTORC1 activity. mTORC1, mechanistic target of rapamycin complex 1; p70S6K, P70S6 kinase 1; ATF4, transcription factor 4; GCN2, general control non-derepressible 2; eIF2a, eukaryotic translation initiation factor 2A; GβL, G-protein beta-subunit-like.

In the present study, we showed that hsa-miR-663a, which is induced by the GCN2/ATF4 pathway under leucine deprivation, can decrease p70S6K phosphorylation (Figure 4). We also demonstrated that hsa-miR-663a responds to leucine deprivation in an ATF4 signal-dependent manner and can regulate mTORC1 activity and subsequent signaling. The findings provide evidence that hsa-miR-663a could be a novel effector between GCN2 and mTORC1. Collectively, the present omics-based study, for the first time, shows that the GCN2 and mTORC1 pathways are associated *via* miRNA profile and concurrently act under leucine deprivation to regulate the AA metabolism in human hepatocytes.

## Data availability statement

The datasets presented in this study can be found in the NCBI’s Gene Expression Omnibus (GEO) database with accession number GSE208228.

## Author contributions

JY, HJ, and HK designed the experiments. JY performed the experiments. JY, SM, HJ, and HK interpreted and analyzed the data. JY, SM, and HJ wrote the manuscript. HK supervised the research and reviewed the manuscript. All authors have read, edited, and approved the manuscript.

## Footnotes

1
https://bioinfo.ut.ee/primer3/

2
https://www.targetscan.org/vert_70/

## Abbreviations

3 ′-UTR: 3 ′ -untranslated region
AA: amino acid
ATF4: activating transcription factor 4
DMEM: Dulbecco’s modified Eagle’s medium
EAA: essential amino acid
GCN2: general control non-derepressible 2
miRNA: microRNA
mTORC1: mechanistic mammalian target of rapamycin complex 1
PCR: polymerase chain reaction
PRAS40: proline-rich Akt1 substrate of 40 kDa
SE: standard error
siRNA: small interference RNA.
