# Supplementary material for: Activating transcription factor 4-dependent hsa-miR-663a transcription mediates mTORC1/p70S6K1 signaling underleucine deprivation
Source: Front Nutr. 2022 Aug 5;9:965771. doi: 10.3389/fnut.2022.965771 (PMC9389164; doi:10.3389/fnut.2022.965771)

Supplementary Figure

Figure S1


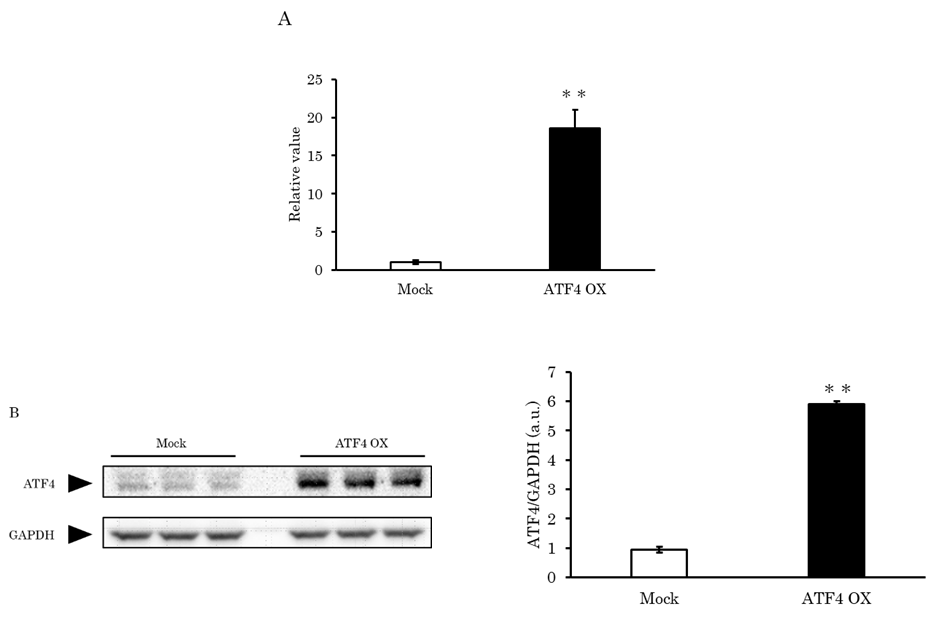


**Figure S1.** Confirmation of ATF4 overexpression in HepG2 cells

(A) HepG2 cells were transfected with pcDNA3.1-ATF4 or MOCK for 24 h and (B) analyzed by western blotting for ATF4 and GAPDH protein levels. Data are mean ± SE. ***p* < 0.01 by t-test.

Supplementary Tables

**Table S1.** Top 5 target genes of the miRNAs predicted by TargetScan Human 7.0

PRAS40, proline-rich Akt substrate of 40 kDa, FAS, fatty acid synthase, SLC25A1, solute carrier family 25 member 1, HRAS, harvey rat sarcoma virus, GLO1, glyoxalase 1, TMSB10, thymosin beta 10, E2F7, E2F transcription Factor 7, NDRG, N-myc downstream regulated 1, SDF4, stromal cell derived factor 4.


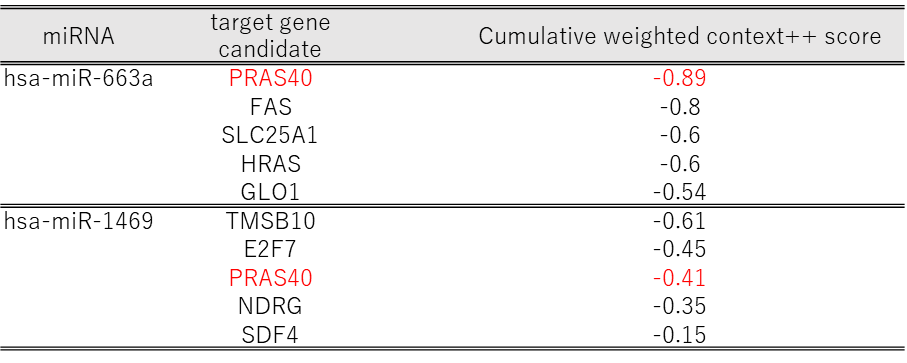


**Table S2.** The predicted hsa-miR-663a and hsa-miR-1469 consensus sequences in PRAS40 3'-UTR. Consensus sequences (|||||) were extracted by referring TargetScan Human 7.0.


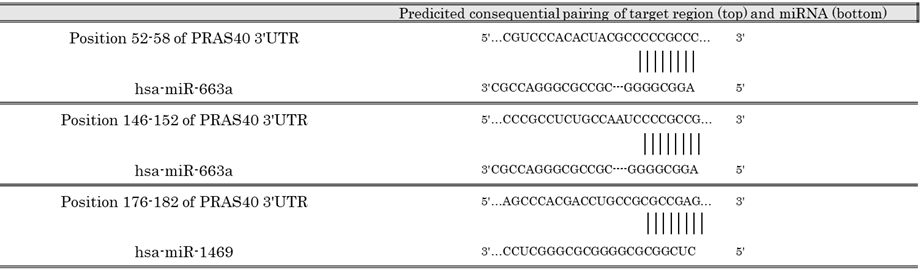

Supplement: Supplementary file 1 [file Table_1.DOCX]
